# Attenuation of ataxia telangiectasia mutated signalling mitigates age‐associated intervertebral disc degeneration

**DOI:** 10.1111/acel.13162

**Published:** 2020-06-21

**Authors:** Yingchao Han, Chao‐Ming Zhou, Hongxing Shen, Jun Tan, Qing Dong, Lei Zhang, Sara J. McGowan, Jing Zhao, Gwendolyn A. Sowa, James D. Kang, Laura J. Niedernhofer, Paul D. Robbins, Nam N. Vo

**Affiliations:** ^1^ Department of Spine Surgery Renji Hospital School of Medicine Shanghai Jiao Tong University Shanghai China; ^2^ Department of Spine Surgery Shanghai East Hospital School of Medicine Tongji University Shanghai China; ^3^ Ferguson Laboratory for Orthopedic and Spine Research Department of Orthopedic Surgery University of Pittsburgh Pittsburgh Pennsylvania; ^4^ Department of Molecular Medicine Center on Aging The Scripps Research Institute Jupiter Florida; ^5^ Institute on the Biology of Aging and Metabolism and Department of Biochemistry, Molecular Biology and Biophysics University of Minnesota Medical School Minneapolis Minnesota; ^6^ Department of Physical Medicine and Rehabilitation University of Pittsburgh School of Medicine Pittsburgh Pennsylvania; ^7^ Department of Orthopedics Brigham and Women's Hospital School of Medicine Harvard University Boston Massachusetts

**Keywords:** accelerated ageing, ATM, endogenous DNA damage, intervertebral disc degeneration, NF‐κB, ROS

## Abstract

Previously, we reported that persistent DNA damage accelerates ageing of the spine, but the mechanisms behind this process are not well understood. Ataxia telangiectasia mutated (ATM) is a protein kinase involved in the DNA damage response, which controls cell fate, including cell death. To test the role of ATM in the human intervertebral disc, we exposed human nucleus pulposus (hNP) cells directly to the DNA damaging agent cisplatin. Cisplatin‐treated hNP cells exhibited rapid phosphorylation of ATM and subsequent increased NF‐κB activation, aggrecanolysis, decreased total proteoglycan production and increased expression of markers of senescence, including p21, γH_2_AX and SA‐ß‐gal. Treating cisplatin‐exposed hNP cells with an ATM‐specific inhibitor negated these effects. In addition, genetic reduction of ATM reduced disc cellular senescence and matrix proteoglycan loss in the progeroid *Ercc1^−/∆^* mouse model of accelerated ageing. These findings suggest that activation of ATM signalling under persistent genotoxic stress promotes disc cellular senescence and matrix homeostatic perturbation. Thus, the ATM signalling pathway represents a therapeutic target to delay the progression of age‐associated spine pathologies.

## Graphical abstract

Persistent DNA damage causes chronic activation of ATM signaling, leading to disc cellular senescence and degeneration. Attenuation of ATM signaling mitigates disc cellular senescence and intervertebral disc degeneration.

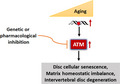

## 1 INTRODUCTION

Older adults commonly experience age‐associated chronic diseases (Christensen, Doblhammer, Rau, & Vaupel, 2009), including musculoskeletal disorders of the spine, which are increasingly prevalent and contribute significantly to disability in the elderly (Goldring and Goldring, (2007)). Indeed, a common sequela of spinal disorders is low back pain, a condition cited as the second most common reason for a doctor's visit in the United States, costing more than $100 billion in direct and indirect costs (Luoma et al., 2000).

Intervertebral disc degeneration (IDD) is a leading contributor in musculoskeletal disorders of the spine, with ageing being a primary risk factor of IDD (Miller, Schmatz, and Schultz (1988); Vo, 2016). Typical age‐associated IDD changes include loss of extracellular matrix proteoglycans (PGs) and hydration leading to tissue fissures and disc height loss (Vo, 2016). These degenerative changes are most likely the result of the loss of functional disc cells required for maintaining matrix homeostasis. Intervertebral discs (IVDs) consist primarily of two types of cells residing within an extensive extracellular matrix network of primarily collagens and proteoglycans. Fibrochondrocytes are found in the outer, ligamentous annulus fibrosus (AF), while chondrocyte‐like cells reside in the inner, gelatinous nucleus pulposus (NP) of the disc (Hunter, Matyas, and Duncan (2004)). Mostly avascular, the disc is sparsely populated by these cells, which further underscore their critical role in maintaining matrix homeostasis needed for disc structure and function.

It is now well established that persistent DNA damage causes organismal ageing. DNA damage that is not repaired can induce irreversible cell growth arrest, resulting in cellular senescence and death (Sedelnikova et al., 2004; Zhan, Suzuki, Aizawa, Miyagawa, & Nagai, 2010). Previously, we reported that the DNA repair‐deficient *Ercc1* ^−/^ *^∆^* mouse model of progeria exhibits premature onset of disc ageing, including loss of matrix proteoglycan, reduced disc height and increased cellular senescence (Vo et al., 2010). Potent genotoxic stressors such as ionizing radiation and tobacco smoking also dramatically accelerate similar degenerative disc changes in mice (Nasto, Wang, et al., 2013; Wang, Wang, et al., 2012). These studies suggest that persistent DNA damage promotes loss of functional disc cells by inducing cellular senescence and diminishing their capacity to maintain matrix PG homeostasis. However, how persistent DNA damage mechanistically causes loss of functional disc cells leading to age‐related IDD has not been carefully defined.

Ataxia telangiectasia mutated (ATM) signalling is a major pathway cells utilize to respond to damage to the genome, which is constantly under assault by both endogenous and environmental factors. Ataxia telangiectasia mutated is a serine–threonine kinase that belongs to the evolutionary conserved phosphatidylinositol‐3‐kinase‐related protein kinase family. Ataxia telangiectasia mutated kinase is required to recruit multiprotein complexes to the site of DNA damage during the DNA damage response (DDR) (Shiloh,^2003^). During this recruitment, the activated ATM kinase phosphorylates various proteins, including p53, histone H2AX (Histone variant of the canonical histone H2A) and checkpoint kinase CHK2 (Checkpoint kinase that regulates cell cycle), to coordinate arrest of the cell cycle, repairing DNA and/or inducing apoptosis (Bakkenist & Kastan,^2003^). Hence, ATM is a central mediator of DDR signalling. Moreover, persistent activation of DDR/ATM signalling in human fibroblasts has been reported to trigger cellular senescence (Fumagalli, Rossiello, Mondello, & d’Adda di Fagagna,^2014^; Rodier etal.,^2009^). However, the role of ATM signalling in modulating DNA damage‐induced cellular senescence and other degenerative changes in the spine has yet to be investigated.

Increased cellular senescence in degenerating discs represents a potential mechanism by which disc tissue loses its ability to regulate matrix homeostasis. Persistent DNA damage induces cellular senescence, the state in which cells undergo irreversible growth arrest but remain metabolically active (d'Adda di Fagagna, 2008; van Deursen, 2014). Senescent cells also can acquire a phenotype known as the senescence‐associated secretory phenotype (SASP) (Coppe et al., 2008) whereby they secrete certain inflammatory cytokines and matrix metalloproteinases (MMPs). Recent studies report that senescent disc cells also exhibit SASP and a reduced ability to produce matrix (Dimozi et al., 2015; Ngo et al., 2017). Accumulation of senescent cells can impair tissue regeneration and homeostasis, leading to metabolic dysfunction and a variety of diseases characterized by accelerated ageing of one or more organ systems (Hasty, Campisi, Hoeijmakers, van Steeg, & Vijg, 2003; van Deursen, 2014). Indeed, clearance of senescent cells using pharmacologic or genetic strategies leads to an extension of health span and lifespan (Baker et al., 2016; Chang et al., 2016; Zhu et al., 2015).

In the present study, we tested our working hypothesis that persistent unrepaired DNA damage leads to chronic dysregulated activation of ATM signalling, driving NF‐κB activation, disc cellular senescence and matrix homeostatic perturbation. We demonstrated that persistent DNA damage‐activated ATM signalling is closely correlated with elevated disc cellular senescence and disc matrix catabolism. Moreover, genetic and chemical inhibition of ATM signalling mitigates cellular senescence and other age‐associated degenerative changes in DNA repair‐deficient *Ercc1* ^−/^ *^∆^* mice and in a human disc cell culture model of genotoxic stress.

## 2 RESULTS

### 2.1 Establishment of the cell model of genotoxic stress‐induced disc degeneration

**Figure 1:**
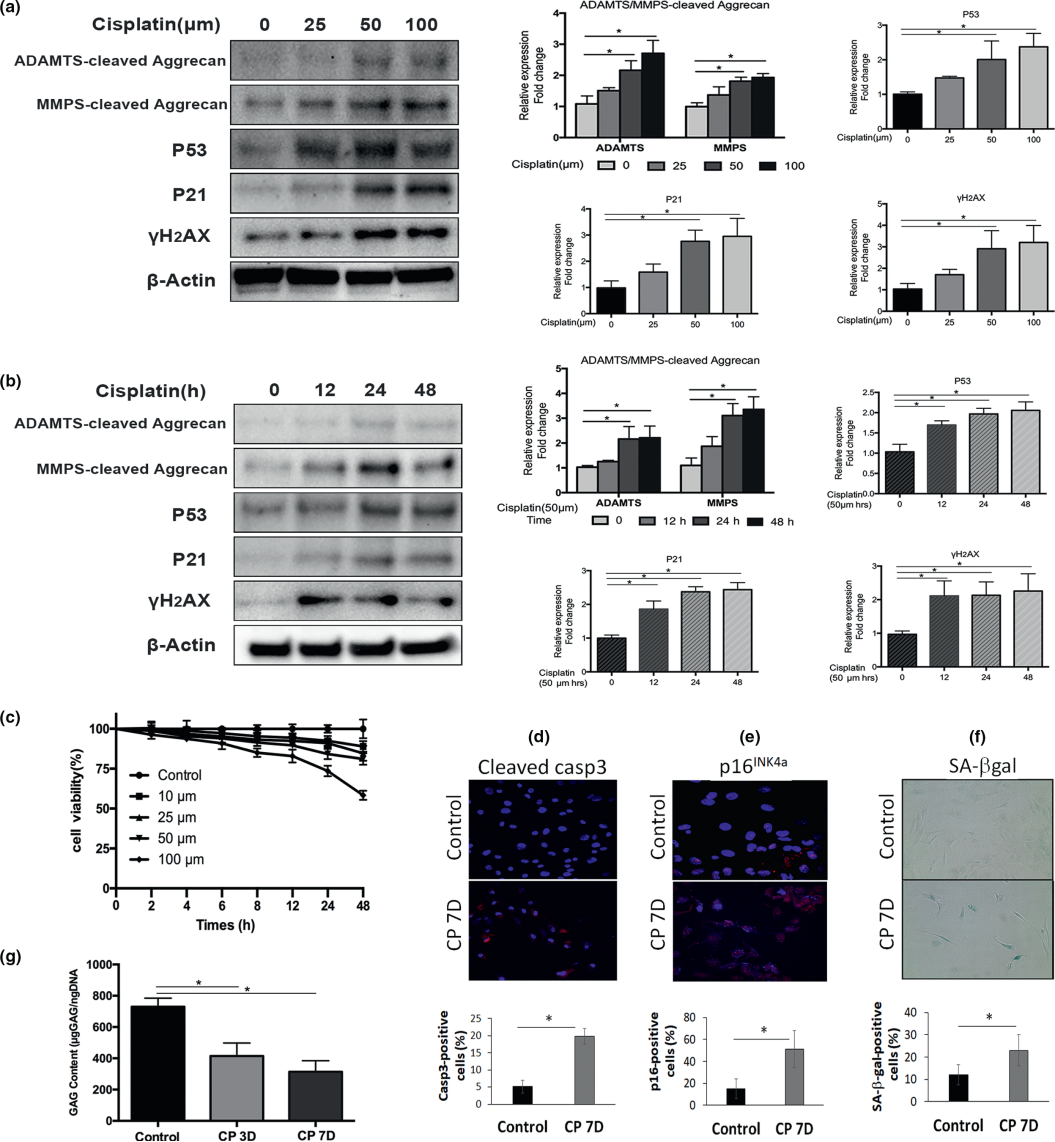
Cisplatin exposure induces cellular senescence and matrix catabolism in human nucleus pulposus cells. Western blot analyses of human nucleus pulposus (hNP) cells treated with different doses (a) and durations (b) of cisplatin for the senescence markers p53, p21 and γH2AX and for the G1 containing aggrecan fragments from the matrix metalloproteinases (MMP)‐ and ADAMTS‐mediated proteolytic cleavage within the interglobular domain (IGD) of aggrecan. Aggrecan fragments shown were generated from MMP‐mediated cleavage (~55kDa) and ADAMTS‐mediated cleavage (~65kDa) of aggrecan IGD. Protein levels were normalized against β‐actin, and graph shows average values ± *SD*. (*n* = 3), *p < .05 versus. control. (c) Cell viability was determined by the CCK‐8 assay on hNP cells treated with different concentrations of cisplatin up to 48 hr. The number of viable cells decreased in a dose‐ and time‐dependent manner. (d) Apoptosis was assayed by cleaved caspase 3 immunofluorescence. (e) Cellular senescence was assayed by p16^INK4a^ immunofluorescence. (f) SA‐β‐gal activity staining (100x magnification) of hNP cell culture treated with cisplatin for 24 hr and then returned to normal media for 7 days. Graph shows the percentage of SA‐β‐gal‐positive cells. (g) Dimethylmethylene Blue assay for total glycosaminoglycan of hNP cells treated with cisplatin for 24 hr and then returned to normal media for 3 days (CP3D) and 7 days (CP7D)

At the cellular level, time‐dependent accumulation of stochastic damage to macromolecules, including DNA, is thought to drive age‐related decline in organ function. To mimic disc DNA damage in vivo, cultures of human NP cells were exposed to cisplatin to cause DNA damage, including interstrand crosslinks, which lead to double‐stranded breaks (DSBs). The level of the phosphorylated histone H2AX variant (γH_2_AX), a marker for DSBs, was increased in cells exposed to cisplatin (Figure 1a and 1b).

To determine the regimen of cisplatin treatment that induces genotoxic stress without significant cell death, cell viability was measured using the CCK‐8 assay following treatments of Human nucleus pulposus (hNP) cell cultures with different cisplatin concentrations and durations. During a 48‐hr exposure, hNP cell viability remained robust at low (10–50 μM), but not high (100 μM) concentrations of cisplatin. For instance, 10 μM cisplatin caused a 10% reduction in hNP cell viability, while 100 μM cisplatin caused a 40% decrease in viability after 48 hr of exposure (Figure 1c). 25–50μM cisplatin was chosen for the subsequent experiments because cellular senescence, as assessed by γH_2_AX and p53 and p21 markers, but not cell viability, was significantly affected at these cisplatin concentrations (Figure 1b and 1c).

### 2.2 Cisplatin exposure induces cellular senescence and suppresses matrix PG production in hNP cell culture

Human nucleus pulposus cells exposed to 50 μM cisplatin for 24 hr, then incubated for an additional 3 or 7 days in media without cisplatin, had a twofold reduction in glycosaminoglycan (GAG) content compared to untreated control cells (Figure 1g). Seven days post‐treatment, 50% of the cisplatin‐treated hNP cells stained positive for the cellular senescence marker p16^INK4a^ (Figure 1e) and exhibited a twofold increase compared to untreated cells in the number of cells staining positively for senescence‐associated β‐galactosidase (SA‐β‐gal) activity (Figure 1f). Expression of other cell senescent markers, p53 and γH_2_AX, increased in hNP cells treated with increasing cisplatin doses (Figure 1a) and exposure duration (Figure 1b). An important target of p53 is p21^Cip1^, a cyclin‐dependent kinase inhibitor that causes cell growth arrest and senescence. p21^Cip1^ was also upregulated in cisplatin‐treated hNP cells (Figure 1a). Additionally, 20% of the cisplatin‐treated hNP cells stained positive for cleaved caspase 3, a marker of apoptosis, 7 days post‐treatment (Figure 1d). These results demonstrate that cisplatin induces cellular senescence as well as apoptosis in hNP cells.

### 2.3 Cisplatin exposure induces aggrecanolysis in hNP cell culture

Aggrecan is a major extracellular matrix proteoglycan in disc NP tissue. Aggrecanolysis, proteolytic fragmentation of aggrecan, is an important marker of disc matrix catabolism and perturbed PG homeostasis. To investigate the effects of DNA damage on matrix catabolism in disc cells, aggrecanolysis was measured by Western blot in hNP cell cultures exposed to increasing cisplatin concentrations (0–100 μM) for 24 hr (Figure 1a) or increasing time durations (0–48 hr) at 50 μM cisplatin (Figure 1b). Proteolytic cleavage within the interglobular domain of aggrecan is considered most pathological, as this results in the loss of the entire GAG attachment region essential for the biomechanical properties of aggrecan in cartilaginous tissue such as the intervertebral discs (Roughley, Alini, & Antoniou, 2002; Wang, Nasto, et al., 2012). Levels of G1‐containing aggrecan fragments resulting from MMP‐ and a disintegrin and metalloproteinase with thrombospondin motifs (ADAMTS)‐mediated cleavage of the interglobular domain of aggrecan increased with time and dose of cisplatin treatments (Figure 1a and 1b). These results demonstrate that disc cells exposed to genotoxic stress undergo catabolic responses leading to aggrecanolysis.

### 2.4 Cisplatin exposure activates ATM and NF‐κB signalling

**Figure 2:**
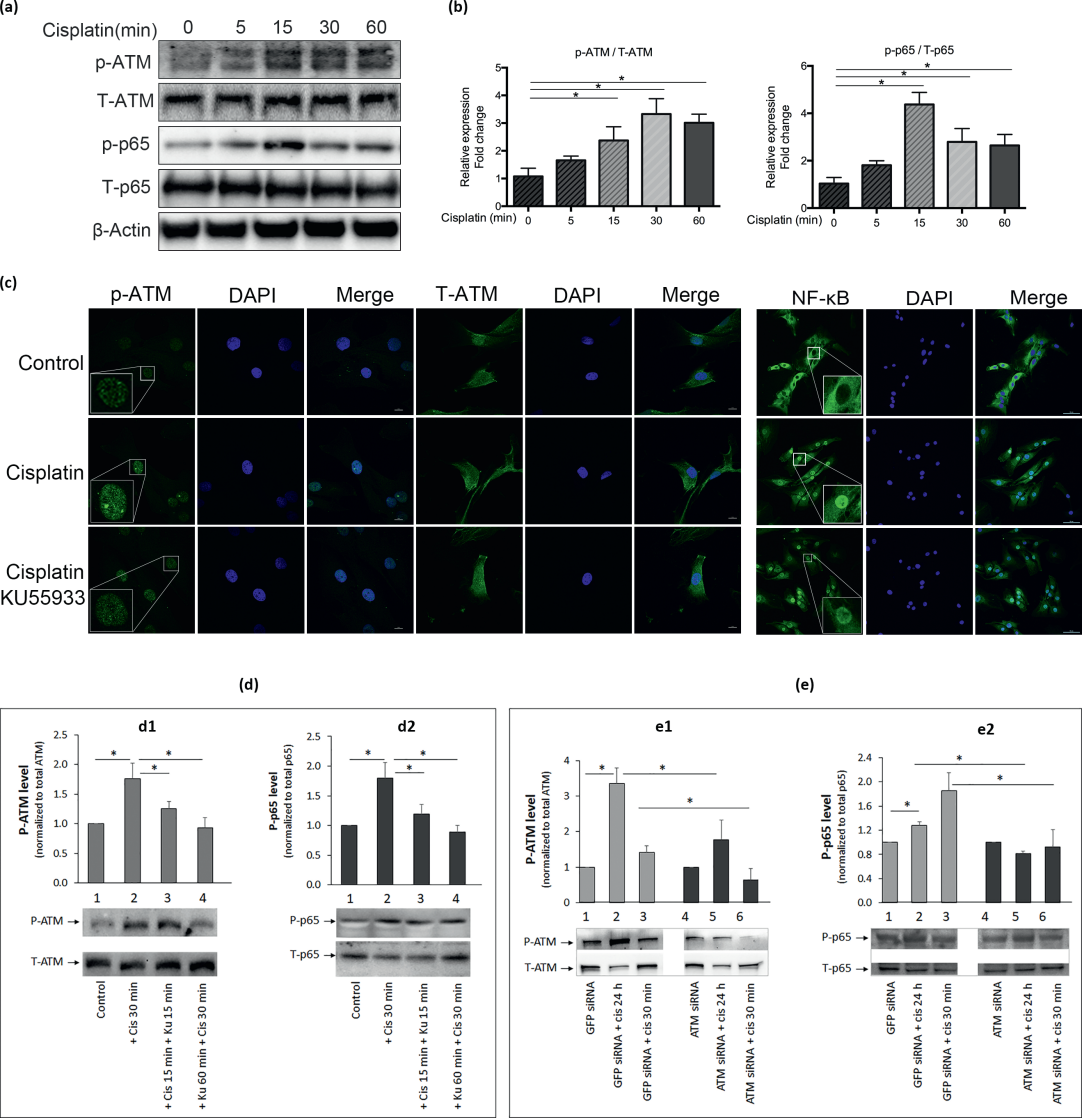
Cisplatin treatment activates ataxia telangiectasia mutated (ATM) and NF‐κB signalling in human nucleus pulposus (hNP) cells. Rapid activation of ATM and NF‐κB signalling in hNP cells following cisplatin exposure. (a) Western analysis of cell extracts following treatment of hNP cell culture with 50μM cisplatin for 0–60 min. (b) Activation of ATM and NF‐κB was assessed by measuring the levels of phosphorylated ATM (p‐ATM) and p65 (p‐p65) proteins, respectively, and normalizing to total ATM (T‐ATM) and total p65 (T‐p65) protein. β‐actin was used as a loading control. (c) Independent confirmation by immunofluorescence detection of cisplatin‐induced activation of ATM and NF‐κB signalling in hNP cells. Human nucleus pulposus cells incubated with or without 50μM cisplatin 30 min were immunostained for p‐ATM, total ATM (T‐ATM) and p65 (NF‐κB; green) and counterstained with DAPI (blue) to stain nuclei. NF‐κB activation was assessed by p65 nuclear translocation from the cytoplasm. p65 mostly resides in the cytoplasm of untreated (control) and nuclei of cisplatin‐treated hNP cells. In hNP cell cultures treated with both cisplatin and the ATM inhibitor KU55933, the level of nuclear p65 immunostaining decreased compared to cisplatin‐treated hNP cells. Images were taken at 400X. Scale bars, 10μm. Quantitative Western blots of p‐ATM (d) and p‐p65 (e) proteins in cell extracts of hNP cells treated with 50μM cisplatin for 30 min with 2μM KU55933 or ATM siRNA. Panel D also showed the effects of adding KU55933 before and after cells were treated with cisplatin. Both KU55933 and ATM siRNA treatment blunted cisplatin‐induced activation of ATM and NF‐κB. Graph shows average values ± *SD*. (*n* = 4), * *p* < .05 versus. control

Autophosphorylation of ATM kinase at Ser1981 is a hallmark of ATM signalling activation and the DNA damage response (DDR). To investigate DDR in cisplatin‐treated hNP cells, we performed a time‐course experiment examining ATM phosphorylation. Treatment of human NP cell culture with 50μM cisplatin induced phosphorylation of ATM kinase at its Ser1981 within 5 min, with the phosphorylation level peaking after 30 min of treatment (Figure 2a and 2b). However, the same treatment did not influence total ATM kinase protein level. This result was confirmed by immunofluorescence detection of p‐ATM (Figure 2c) as well as using a selective inhibitor of ATM kinase (KU55933), which decreased cisplatin‐induced ATM kinase phosphorylation by 40% (Figure 2d.1). Downregulation of ATM using an siRNA also was performed to independently verify the specificity of KU55933 (Figure 2e.1); both approaches yielded similar suppression of p‐ATM. Phosphorylation and activation of H2AX, a known substrate of p‐ATM kinase, were also increased in cisplatin‐treated hNP cells (Figure 1a and 1b). Together, these results demonstrate that genotoxic stress activates ATM signalling in disc cells.

Under genotoxic stress, ATM has been shown to activate NF‐κB through a nuclear‐initiated pathway (Miyamoto, 2011). NF‐κB signalling plays a vital role in mediating cellular response to stress, but chronic NF‐κB activation has been reported to cause age‐related disorders, including IDD (Nelson, Kucheryavenko, Wordsworth, & von Zglinicki, 2017). In hNP cells, cisplatin treatment rapidly and transiently induced phosphorylation of p65, a subunit of NF‐κB, without affecting the total p65 protein expression. Increased p65 phosphorylation was detected within 5 min of cisplatin exposure, peaked at 15 min, and declined after 30 min (Figure 2a and 2b). Ataxia telangiectasia mutated kinase inhibition by KU55933 (Figure 2c and 2d.2) or ATM siRNA (Figure 2e.2) led to a modest, but significant decrease in NF‐κB activation in cisplatin‐treated hNP cells, suggesting that NF‐κB activation by genotoxic stress in disc cells is mediated, at least in part, by the ATM pathway. Together, these results demonstrate that hNP cells responded to cisplatin by inducing DDR involving activation of ATM and NF‐κB signalling pathways.

### 2.5 Blocking ATM activity reduces cellular senescence and improves matrix proteoglycan production in cisplatin‐treated hNP cells

**Figure 3:**
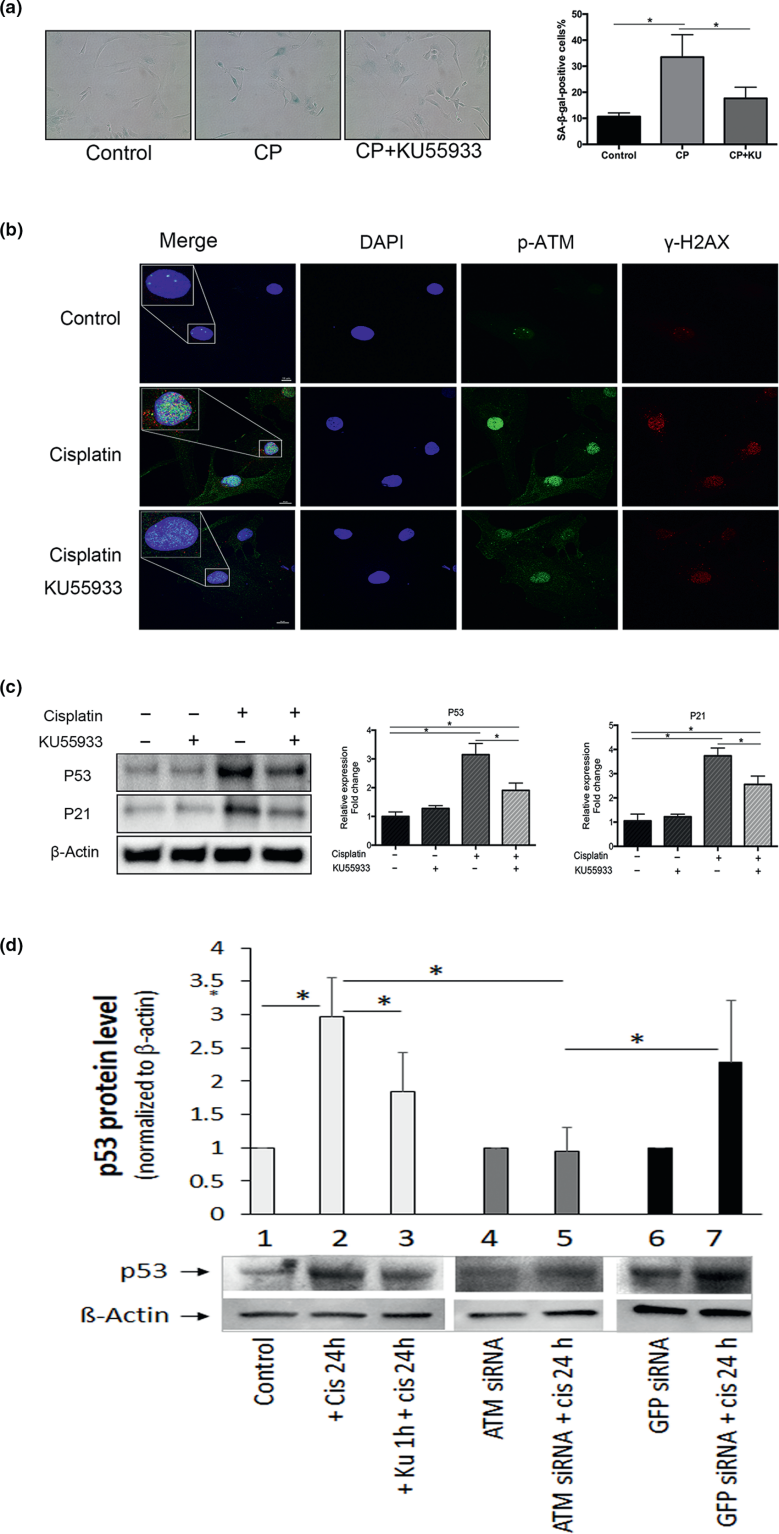
Effects of the ataxia telangiectasia mutated (ATM) inhibition on cisplatin‐induced human nucleus pulposus (hNP) cellular senescence. (a) SA‐β‐gal activity staining of hNP cell cultures treated with cisplatin with or without KU55933 for 24 hr followed by 6 days in drug‐free media. Graph shows the percentage of SA‐β‐gal‐positive cells. (b) KU55933 treatment decreased cisplatin‐induced p‐ATM (green) and γH2AX (red) immunofluorescence in hNP cells treated with cisplatin with or without KU55933 for 60 min. DAPI, nuclear stain (blue). Images were taken at 400X. Scale bars, 10μm. (c) Western analysis for senescence markers (p53, p21) in extracts from hNP cells treated with 50μM cisplatin for 24 hr with 2μM KU55933 or ATM siRNA. The protein levels were normalized against β‐actin, and graph shows average values ± *SD*. (*n* = 4), * *p* < .05 versus. control

An ATM‐specific inhibitor was used to interrogate whether ATM mediates genotoxic stress‐induced disc senescence and matrix PG loss. Human nucleus pulposus cell cultures were treated with the ATM inhibitor KU55933 for 1 hr before being exposed to 50 μM cisplatin for 24 hr, followed by 6 days of incubation in drug‐free media. Under this treatment regimen, overall cellular senescence decreased, as evidenced by a reduction in the number of SA‐β‐gal‐positive cells from 33% to 18% (Figure 3a) and decreased levels of the senescence markers p53 and p21 (Figure 3c). In addition, immunofluorescence detection of p‐ATM and γH2AX as well as co‐localization of these two proteins in cisplatin‐treated hNP cells was reduced by KU55933 treatment (Figure 3b).

**Figure 4:**
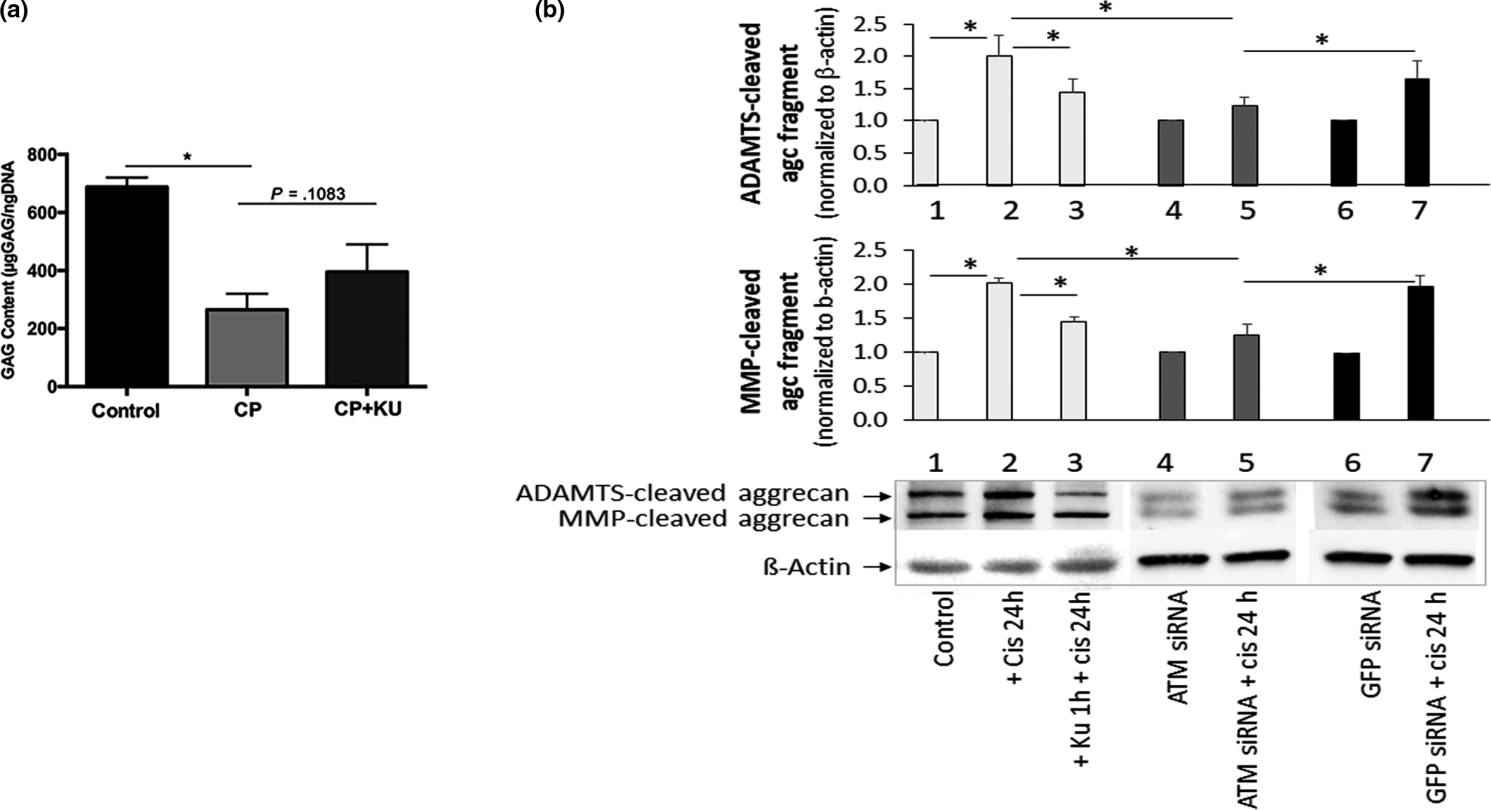
Effects of the ataxia telangiectasia mutated (ATM) inhibition on cisplatin‐induced disc cell matrix proteoglycan content and integrity. (a) Dimethylmethylene Blue assay for total glycosaminoglycan (GAG) produced by human nucleus pulposus (hNP) cells treated with cisplatin with or without KU55933 for 24 hr followed by 3 days in drug‐free media. KU55933 treatment improved GAG content in cisplatin‐treated hNP cells. (b) Western analysis for aggrecan fragments in extracts from hNP cells treated with 50μM cisplatin for 24 hr with 2μM KU55933 or ATM siRNA. The protein levels were normalized against β‐actin, and graph shows average values ± *SD*. (*n* = 4), * *p* < .05 versus. control

We also found that KU55933 partially reversed cisplatin‐induced suppression of GAG production in hNP cells (Figure 4a). Additionally, the increased levels of ADAMTS‐ and MMP‐cleaved aggrecan fragments caused by 25 µM cisplatin decreased upon ATM inhibition either by KU55933 or ATM siRNA (Figure 4b), suggesting that cisplatin‐induced aggrecan fragmentation is mediated, in part, via the ATM pathway. Taken together, these results suggest that ATM signalling mediates the effects of DNA damage on proteoglycan matrix imbalance and cellular senescence in hNP cells.

### 2.6 ROS is involved in ATM activation by cisplatin

**Figure 5:**
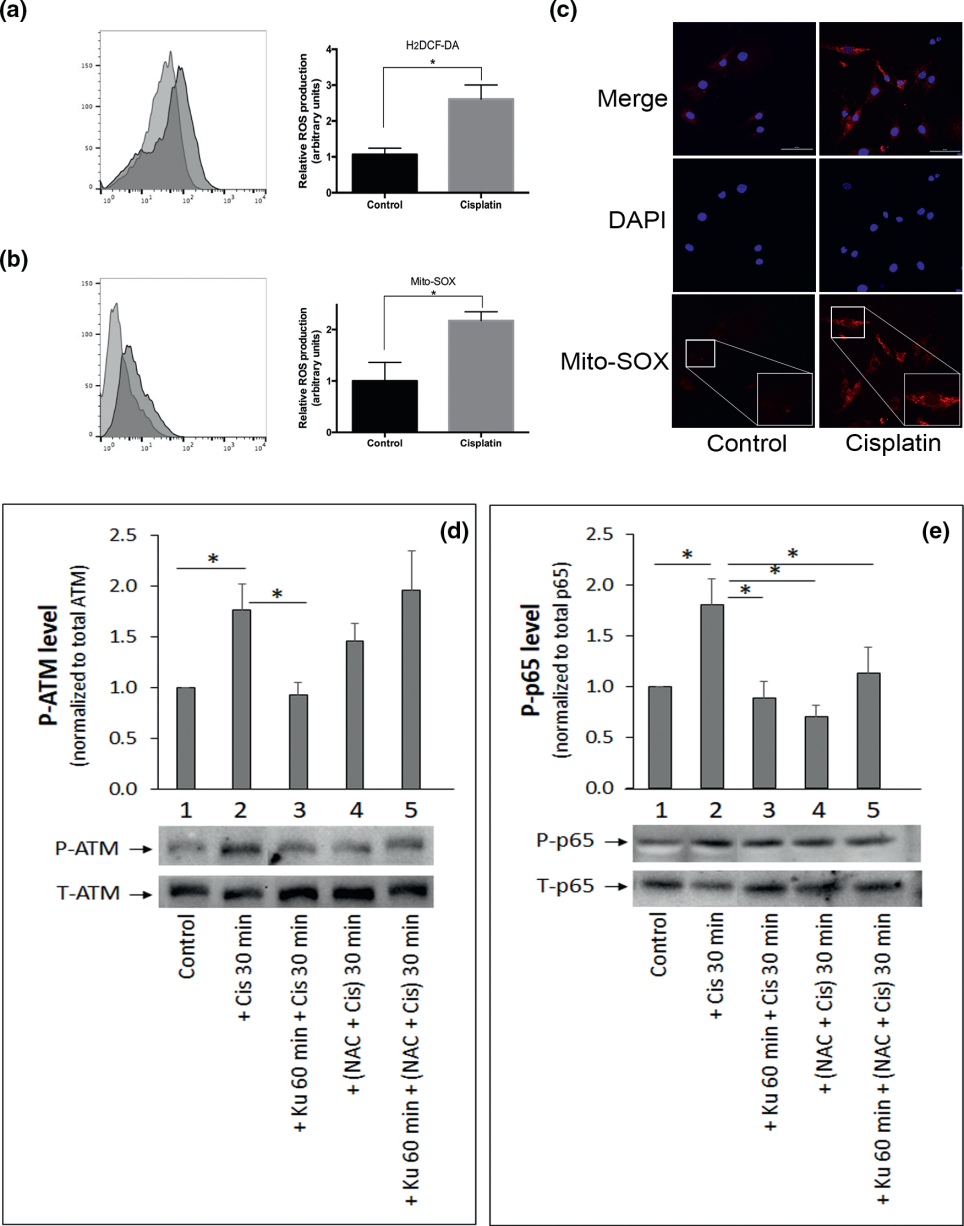
Cisplatin exposure triggers reactive oxygen species (ROS) production in human nucleus pulposus (hNP) cells. Detection by flow cytometry of DCFH‐DA staining for total intracellular ROS (a) and mitochondria‐generated ROS (b) in hNP cells following 50μM cisplatin treatment for 15 min. (c) Representative images obtained by confocal fluorescence microscopy of MitoSOX Red fluorescence of human NP cells treated with 25μM cisplatin for 15 min. DAPI was used as nuclear stain (blue). Images are taken at magnification 400X; scale bar is 10μm. Effects of the antioxidant N‐acetyl L‐cysteine (NAC), KU55933, or both NAC and KU55933 on blunting cisplatin‐induced activation of ataxia telangiectasia mutated (panel d) and NF‐κB (panel e) as measured by Western analysis of cell extracts of hNP cell cultures treated before or after with 25μM cisplatin for a total of 30 min. Graphs show average values ± *SD*. (*n* = 3), * *p* < .05 versus. control

DNA damage has been reported to promote oxidative stress, which in turn activates ATM signalling (Caputo, Vegliante, & Ghibelli, 2012; Guo, Kozlov, Lavin, Person, & Paull, 2010a). We and others have previously demonstrated a causal role of reactive oxygen species (ROS) in age‐related IDD (Dimozi et al., 2015; Nasto, Robinson, et al., 2013). However, it is still unknown whether DNA damage directly promotes oxidative stress in disc cells. Hence, in the present study we evaluated the overall level of intracellular ROS in hNP cells after they were exposed to 50 μM cisplatin for 15 min (Figure 5a). Using the H_2_DCFDA probe for cytosolic ROS, we observed 2.6 ± 0.2‐fold increase in total ROS following cisplatin treatment. MitoSOX flow cytometry and staining for mitochondrial superoxide radicals showed that the mitochondria contributed significantly to the cisplatin‐induced production of intracellular ROS in hNP cells (Figure 5b and 5c). These data demonstrate that cisplatin induces a substantial level of oxidative stress in human NP cells through mitochondria‐generated ROS.

Chronic activation of NF‐κB signalling by genotoxic and oxidative stress is known to drive pathological ageing, including age‐related IDD (Tilstra et al., 2012). Cisplatin treatment of hNP cells resulted in NF‐κB activation, as evidenced by nuclear translocation of p65, a subunit of NF‐κB, from the cytoplasm (Figure 2c) and increased phosphorylated p65 protein by Western blot analysis (Figure 5e). This activation of NF‐κB is mediated, in part, by cisplatin‐induced ROS production because treatment of hNP cells with 5mM of the ROS scavenger N‐acetyl L‐cysteine (NAC) prior to cisplatin exposure inhibited the phosphorylation of p65 as well as ATM. These results indicate that ROS generated from cisplatin exposure mediates activation of both NF‐κB and ATM signalling in hNP cells (Figure 4d). While NAC or KU55933 individually reduces p‐ATM and p‐p65 expression in cisplatin‐treated hNP cells, treating cisplatin‐exposed hNP cells with both NAC and KU55933 resulted in no synergistic suppression of expression of p‐ATM and p‐p65 (Figure 5d and 5e). In fact, the combined treatment not only failed to show additive or synergic effect, but also brought back the p‐ATM level to the cisplatin‐treated cells. This antagonistic effect might be due to direct binding of NAC to Ku55933 and negating its ATM inhibition or indirectly by NAC modifying another biomolecule that in turn antagonizes Ku55933 inhibition of ATM.

### 2.7 Genetic reduction of ATM reduces disc cellular senescence and matrix proteoglycan loss in the progeroid *Ercc1^−/∆^* mice

The DNA repair‐deficient, progeroid *Ercc1^−/∆^* mice share a remarkable number of important ageing features with old wild‐type mice. These include loss of functional stem cells, accumulation of senescent cells, similar changes in histopathology and genome‐wide gene expression profiles in multiple tissues, and various age‐associated pathologies (Dolle et al., 2011; Gregg, Gutiérrez, et al., 2011; Niedernhofer et al., 2006; Yousefzadeh et al., 2020). In addition, *Ercc1^−/∆^* mice exhibit an accelerated spine ageing phenotype (Tilstra et al., 2012; Vo et al., 2010) that is caused by an impaired ability to repair DNA adducts similar to those caused by cisplatin (Gregg, Robinson, & Niedernhofer, 2011b). Thus, although they are not necessarily identical to natural ageing mice, these accelerated ageing *Ercc1^−/∆^* mice are excellent models to probe the underlying mechanisms of age‐related pathologies, including intervertebral disc degeneration.

**Figure 6:**
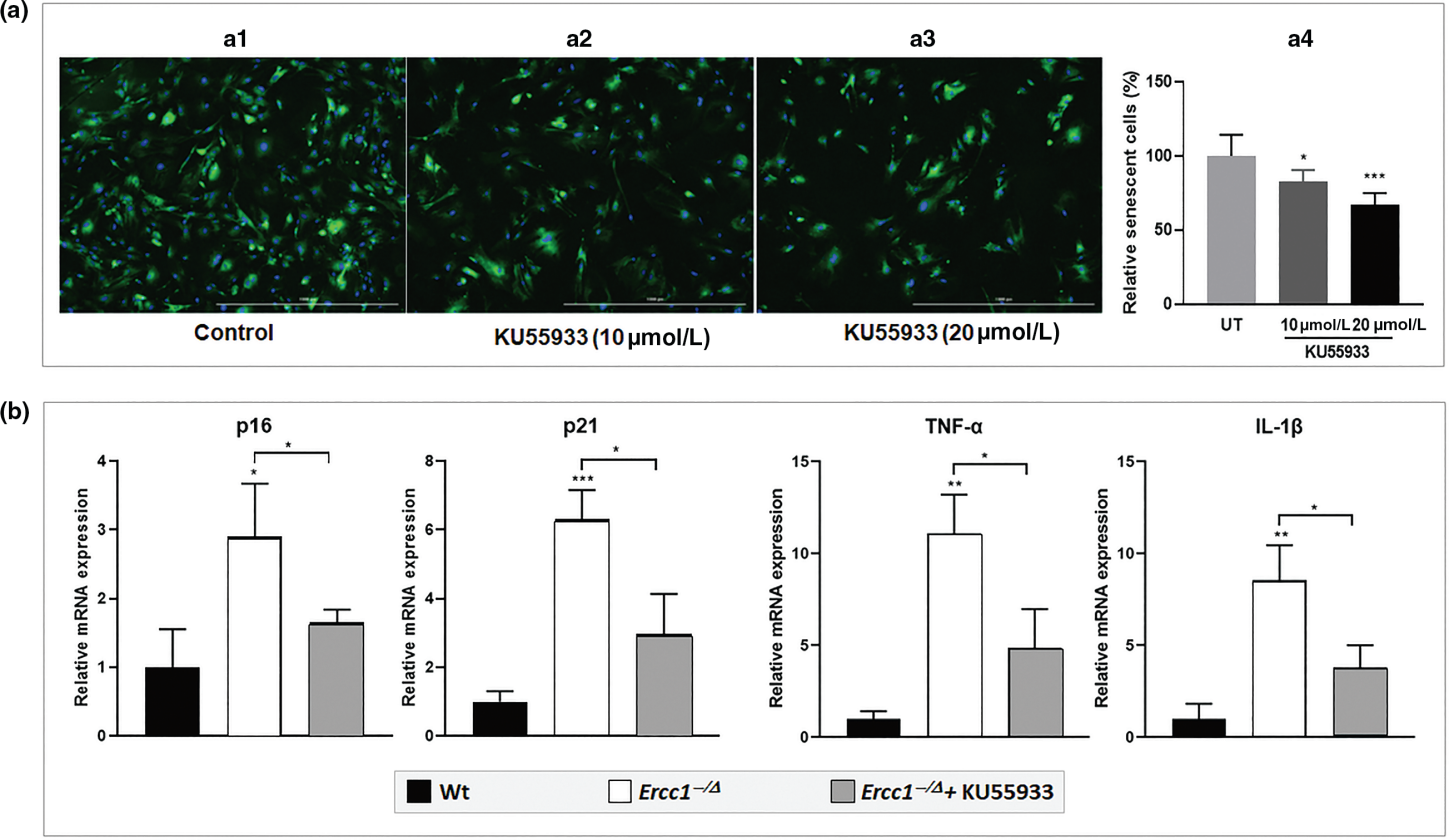
Effects of the ataxia telangiectasia mutated inhibition on *Ercc1^−/−^* MEF cellular senescence. (a) Representative images of SA‐β‐gal senescence assay by C_12_FDG staining in senescent *Ercc1^−/−^* MEFs in cell cultures with or without treatment of KU55933, where blue fluorescence indicates DNA staining with Hoechst 33,324 and green fluorescence indicates SA‐β‐gal staining with C_12_FDG. Quantification of C_12_FDG senescence assay is shown in panel a.4. Error bars indicate *SD* for *n* = 3. (b) qRT–PCR analysis of expression of senescence biomarkers in nonsenescent WT and senescent *Ercc1^−/−^* MEFs in cell cultures with or without treatment of KU55933 (10 μM). Error bars indicate *SD* for *n* = 3

The persistent DNA damage in ERCC1‐deficient mice drives cellular senescence in many tissues, (Robinson et al., 2018) including the IVD (Ngo et al., 2017) and mouse embryonic fibroblasts (MEFs) (Figure 6a). To determine whether ATM signalling mediates cellular senescence that is driven by persistent DNA damage in these mice, we first studied the effects of KU55933 on cellular senescence of *Ercc1^‐/‐^* MEF culture model. Indeed, *Ercc1^‐/‐^* MEF cultures treated with the ATM inhibitor KU55933 resulted in an overall decrease in cellular senescence, as evidenced by the KU55933 dose‐dependent reduction in the number of SA‐β‐gal‐positive cells (Figure 6a) and decreased mRNA levels of the senescence markers *p16^Ink4a^* and *p21^Cip1^* (Figure 6b). In addition, KU55933 treatment significantly reduced the expression of the key SASP factors *TNF‐α* and *IL‐1β* (Figure 6b). These findings are consistent with those observed above in our hNP cell culture model treated with cisplatin and KU55933; findings from the two different cell culture models indicate that ATM mediates DNA damage‐induced cellular senescence.

**Figure 7:**
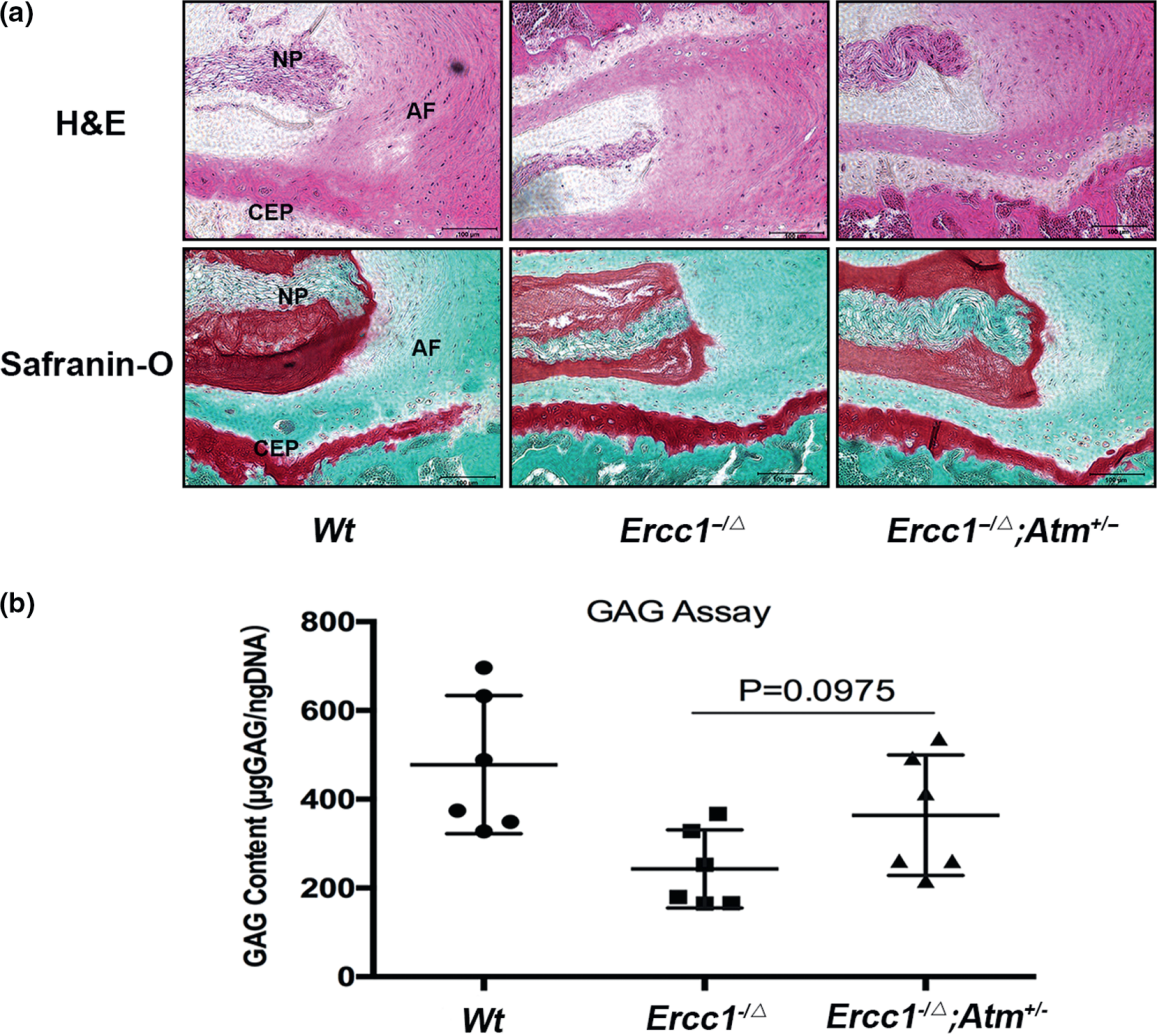
Genetic reduction of ataxia telangiectasia mutated mitigates loss of the disc matrix in *Ercc1^−/∆^* mice. (a) Representative histologic images from sagittal sections of the spines stained with H&E and safranin O/fast green. Greater disc cellularity, both in NP and in AF, and annular matrix organization were observed in *Ercc1^−/∆^*; *Atm^±^* mice compared to *Ercc1^−/∆^* mice. Images were taken at 200X. Scale bars, 200μm. (b) Dimethylmethylene Blue assay revealed improved glycosaminoglycan content of NP tissue in 16‐week‐old *Ercc1^−/∆^*; *Atm^±^* mice compared to *Ercc1^−/∆^* littermates. *N* = 6. Cartilaginous endplate

Next, we determined whether ATM mediates disc cellular senescence and matrix proteoglycan loss in the progeroid *Ercc1^−/∆^* mice through *Atm* genetic depletion. We compared spine degeneration outcome measures between *Ercc1^−/∆^* mice and *Ercc1* ^‐/∆^; *Atm^±^* mice that lacked 1 functional *Atm* allele. Compared to *Ercc1^−/∆^* mice, *Ercc1* ^‐/∆^; *Atm^±^* mice showed increased cellularity in the AF and denser matrix network within the NP, as qualitatively assessed by H&E and safranin O/fast green histological staining (Figure 6a). Additionally, *Ercc1* ^‐/∆^; *Atm^±^* mice showed less AF lamellar disorganization compared to *Ercc1^−/∆^* mice (Figure 7a). Consistent with our histological results, *Ercc1* ^‐/∆^; *Atm^±^* mice exhibited an overall higher level of disc matrix proteoglycan compared to that of *Ercc1^−/∆^* mice, assessed quantitatively by the Dimethylmethylene Blue (DMMB) biochemical assay for total disc GAG content; albeit the difference is not statistically significant (Figure 7b). These results demonstrate that reduction in ATM signalling mitigates disc matrix loss in progeroid *Ercc1* ^−/∆^ mice.

**Figure 8:**
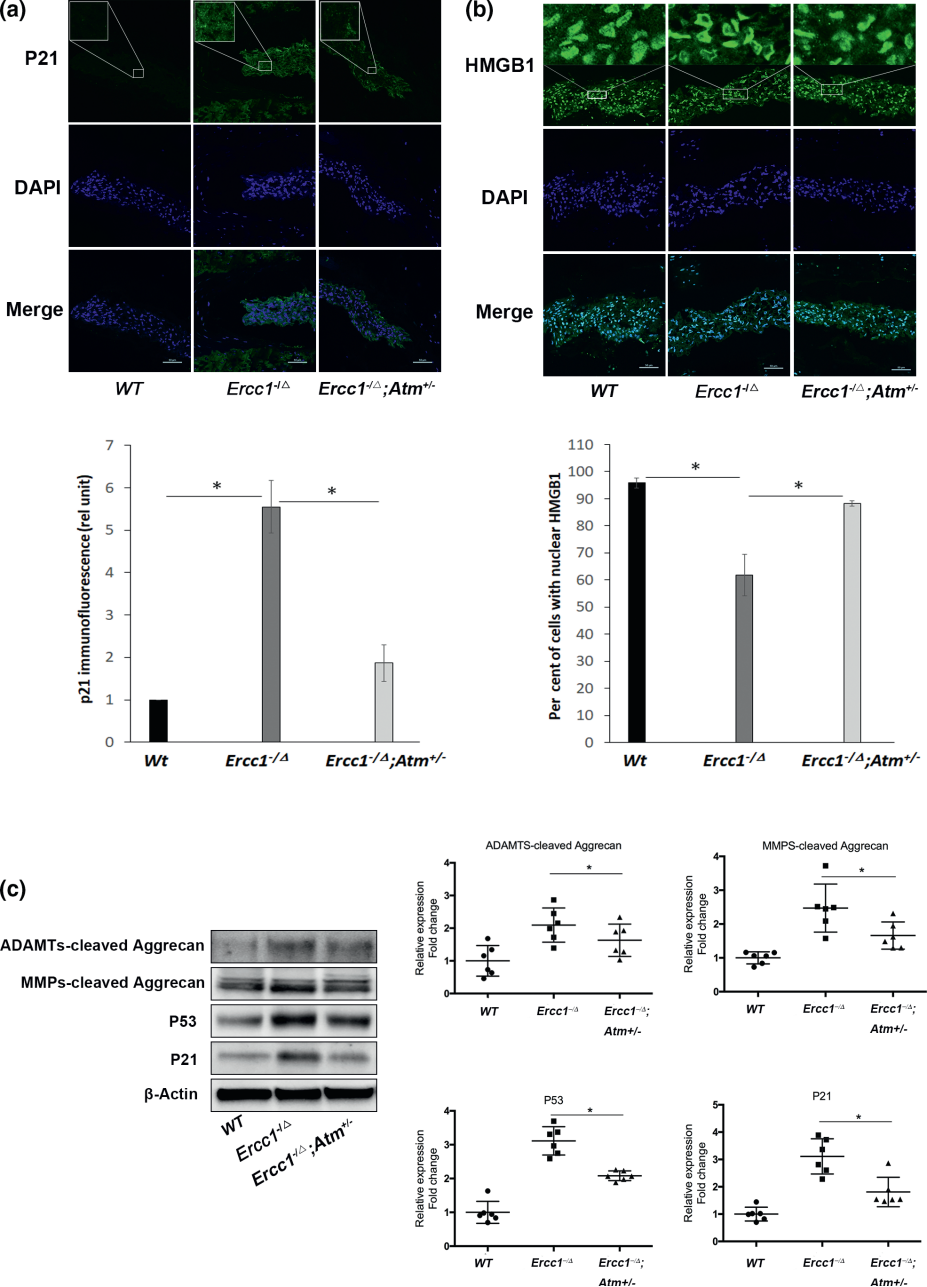
Genetic reduction of ataxia telangiectasia mutated mitigates disc cellular senescence and aggrecanolysis in *Ercc1^−/∆^* mice. Representative images obtained by confocal fluorescence microscopy of the two senescence markers, p21 (a) and HMGB1 (b), in the NP region of the IVD. p21 and HMGB1 (green), DAPI nuclear stain (blue). Reduced p21 immunofluorescence was observed in the disc NP region of *Ercc1^−/∆^*; *Atm^±^* mice compared to *Ercc1^−/∆^* mice. Additionally, nuclear HMGB1 immunofluorescence was mostly absent in the *Ercc1^−/∆^* mice compared to the abundant nuclear HMGB1 in wild‐type and *Ercc1^−/∆^*; *Atm^±^* mice. Images were taken at 400X. Scale bars, 10μm. (c) Western blot analyses of disc tissue extract for the senescence markers p53 and p21 as well as the G1‐containing aggrecan fragments generated from matrix metalloproteinases‐ and ADAMTS‐mediated cleavage within the interglobular domain of aggrecan. All mice were 16‐week‐old at the time of sacrifice. The protein levels were normalized against β‐actin, and graph shows average values ± *SD*. (*n* = 6), * *p* < .05 versus. control

Reduction of ATM signalling also mitigates cellular senescence in *Ercc1^−/∆^* mice in multiple tissues (Jing Zhao et al., 2017). Immunofluorescence analysis of the senescence marker p21^Cip1^ in the disc NP region showed a reduction in *Ercc1* ^‐/∆^; *Atm^±^* mice compared to *Ercc1^−/∆^* mice (Figure 8a). The high mobility group box 1 (HMGB1) protein normally resides in the cell nucleus to modulate gene expression, but in senescent cells, HMGB1 translocates from the nucleus to the cytoplasm and extracellular matrix as an alarmin to signal tissue damage. Thus, cytosolic HMGB1 is an indicator of a stress response frequently observed in senescent cells (Davalos et al., 2013). Immunofluorescence detection of HMGB1 in disc sections showed mostly cytosolic localization in *Ercc1^−/∆^* mice. In contrast, disc HMGB1 in *Ercc1* ^‐/∆^; *Atm^±^* mice and wild‐type mice was localized in the nuclei (Figure 8b). Moreover, immunoblot analysis showed reduced levels of the senescence markers p53 and p21 in discs of *Ercc1* ^‐/∆^; *Atm^±^* mice compared to *Ercc1^−/∆^* mice (Figure 8c). However, disc p53 and p21 levels in *Ercc1^−/∆^*; *Atm^±^* mice were still higher than WT mice, suggesting a single *Atm* allele genetic deletion only partially rescued disc cellular senescence in *Ercc1^−/∆^* mice.

Reduction of ATM signalling also mitigates overall disc matrix catabolism in *Ercc1^−/∆^* mice. Western blot analysis revealed a significant reduction in ADAMTS‐ and MMP‐cleaved disc aggrecan fragments in *Ercc1* ^‐/∆^; *Atm^±^* mice compared to *Ercc1^−/∆^* mice, although the levels of these aggrecan fragments in *Ercc1* ^‐/∆^; *Atm^±^* mice were still higher than those detected in WT mice (Figure 8c). These data suggest that reduction of ATM signalling partially mitigated DNA damage‐induced disc matrix catabolism and cellular senescence in the progeroid *Ercc1^−/∆^* mice.

## 3 DISCUSSION

Persistent DNA damage and the ensuing DNA damage response are implicated as drivers of ageing and age‐related diseases (Shimizu, Yoshida, Suda, & Minamino, 2014; Tilstra et al., 2012). Endogenous and exogenous genotoxins such as ROS and radiation generate DNA damage in living organisms by chemical modification or physical damage (Shimizu et al., 2014). DNA damage is known to drive cellular senescence, which in turn is implicated in driving ageing. Ataxia telangiectasia mutated signalling is a central mediator of cellular response to DNA damage that can lead to cellular senescence (Rodier et al., 2009). Indeed, we recently discovered that *Ercc1* ^‐/Δ^ mice heterozygous for ATM, compared to *Ercc1* ^‐/Δ^ mice, have reduced cellular senescence, improved stem cell function and extended health span (Jing Zhao et al., 2017). Thus, here we tested the hypothesis that unrepaired DNA damage leads to chronic activation of ATM signalling that then drives disc cellular senescence and matrix homeostatic imbalance in the IVD. We demonstrate in the present study that genotoxic stress activates ATM signalling, increases cellular senescence and enhances MMP‐ and ADAMTS‐mediated matrix aggrecan catabolism in a hNP cell culture model. We also show that genetic depletion of ATM mitigates the onset of disc cellular senescence and disc aggrecanolysis in the DNA repair‐deficient *Ercc1^−/∆^* mouse model of accelerated ageing. Altogether, our study demonstrates that chronic activation of ATM signalling mediates degenerative changes in the ageing spine. These findings also suggest that reduction of ATM signalling is a potential therapeutic strategy to minimize spinal degeneration with ageing. However, given the integral role ATM plays in DNA damage response, complete blocking of ATM signalling would disrupt normal cellular homeostasis, likely leading to undesirable consequences. Instead, reduction of the increased level of activation of ATM in older mice and humans should have a beneficial effect.

Persistent DNA damage plays an important role in driving age‐related IDD as demonstrated in several animal model systems (Nasto, Wang, et al., 2013; Vo et al., 2010, 2013; Wang, Wang, et al., 2012). Among the models that best replicates age‐associated pathologies in humans is the DNA repair‐deficient *Ercc1^−/∆^* mouse model of accelerated ageing (Vo et al., 2010). IVDs of these mice exhibit accelerated ageing changes, including elevated cellular senescence and reduced GAG and hydration. However, because DNA repair deficiency is global in the *Ercc1^−/∆^* mice due to whole‐body ERCC1‐XPF depletion, it is not clear whether age‐related IDD in these animals is a result of local or global effects of DNA damage. In other words, the observed increase in disc cellular senescence and GAG loss in *Ercc1* ^−/∆^ mice could be due to the direct effects of DNA damage on disc cells or indirect systemic effects on disc tissue because of DNA damage and/or senescence in other tissues. The fact that cisplatin‐treated hNP cells showed senescence and matrix perturbation suggests that accumulation of DNA damage within disc cells can directly and negatively impact their phenotype and matrix homeostasis.

ATM is a protein kinase that regulates cell cycle progression in response to DSBs (Wu, Shi, Tibbetts, & Miyamoto, 2006). Activation of ATM leads to cell cycle arrest, senescence and/or apoptosis. Conversely, ATM inactivation can reprogram cellular metabolism to overcome replication stress and escape senescence (Bakkenist & Kastan, 2003; Borodkina et al., 2016). Though DNA damage‐induced ATM activation has been established in other tissues, this pathway has not been studied in disc tissue until now. Our current study demonstrates for the first time the role of ATM signalling in modulating DNA damage‐induced cellular senescence and matrix perturbation in the IVD. Treatment of hNP cells with cisplatin resulted in rapid ATM phosphorylation, while inhibition of ATM phosphorylation ameliorated cisplatin‐induced induced cellular senescence and matrix GAG loss in human disc cells. Interestingly, ATM inhibition did not have the anticipated effect of reducing aggrecan fragmentation, suggesting that mechanisms other than ATM signalling mediate DNA damage‐induced aggrecan fragmentation in hNP cells. One possible mechanism is cisplatin‐stimulated ROS production, which can activate NF‐κB, inducing aggrecanolysis through an additional pathway (Figure 4).

Chronic activation of NF‐κB signalling promotes ageing and age‐related pathologies in various tissues, including the IVD (Nasto et al., 2012; Tilstra et al., 2012). Recent studies reveal that genotoxic stress activates NF‐κB via a pathway in which DNA damage‐activated ATM phosphorylates nuclear NEMO leading to its cytoplasmic export to activate IKK, the upstream activator of NF‐κB (Miyamoto, 2011). Moreover, NF‐κB signalling is essential for the establishment of stress‐induced cellular senescence and SASP (Freund, Patil, & Campisi, 2011). NF‐κB knockdown in DNA repair‐deficient *Ercc1^−/∆^* mice significantly ameliorates age‐related disc degeneration (Nasto et al., 2012). Based on these observations, we hypothesized that DNA damage in disc cells activates NF‐κB through ATM signalling leading to cellular senescence and matrix perturbation. However, since cisplatin activates both NF‐κB and ATM, ATM inhibition only modestly blocks cisplatin‐induced NF‐κB activation in hNP cells, and DNA damage also activates NF‐κB through ATM‐independent pathway(s) in hNP cells. Previous studies have shown that DNA damage promotes oxidative stress, which in turn induces oxidative DNA damage in a positive feedback loop (Guo, Deshpande, & Paull, 2010b). Oxidative stress is a well‐established driver of NF‐κB activation and cell senescence. Interestingly, the combined treatment with NAC and KU55993 had no additive or synergistic effect on senescence, suggesting that the senescence is driven predominantly through an ATM‐dependent mechanism induced by oxidative DNA damage. Consistent with this model is the fact that cisplatin‐treated hNP cells produce a large amount of ROS and that blocking ROS production by NAC greatly decreases NF‐κB activation (Figure 4).

In conclusion, our study revealed persistent unrepaired DNA damage in disc cells directly promotes cellular senescence and matrix imbalance, two key features commonly found in ageing and degenerating disc tissue. We also identified and elucidated the role of chronic ATM signalling in driving disc cellular senescence and matrix perturbation in the presence of persistent DNA damage. The ATM‐p53‐p21 axis is a well‐recognized signalling pathway involved in establishing and stabilizing cellular senescence induced by DNA damage (Zhao et al., 2017). Collectively, our findings implicate the ATM‐p53‐p21 signalling pathway as a potential molecular therapeutic target for prevention and treatment of age‐related intervertebral disc degeneration.

## 4 EXPERIMENTAL PROCEDURES

### 4.1 Human NP cell culture

Human nucleus pulposus (hNP) cells were isolated from the disc tissue of eight patients (average age 42.8 ± 3.8; female:male ratio = 6:2) removed during elective surgical procedures for degenerative spinal diseases (degeneration grade 3.1 ± 0.6) (Thompson et al., 1990). Tissue specimens were placed in sterile Ham's F‐12 medium (F‐12; *GibcoBRL*, Grand Island, NY) containing 1% penicillin/streptomycin (P/S; *GibcoBRL*) and 5% foetal bovine serum (FBS; *GibcoBRL*). Human nucleus pulposus tissue was washed 3 times with Hank's balanced salt solution (HBSS; *GibcoBRL*) containing 1% P/S to remove blood and other bodily contaminants prior to isolation. Cells were isolated separately from each specimen as previously described (Moon et al., 2014) and cultured at 37°C, 5% CO2, 5% O_2_ in a humidified incubator. Eight independent hNP passage 1 cultures were plated on 6‐well plates (70%–80% confluence) and treated with cisplatin (*Sigma‐Aldrich,* St. Louis, MO), a chemotherapeutic drug that causes DNA interstrand‐crosslink lesions. Cells were treated at different cisplatin concentrations (0–100 µM) and durations (0–48 hr). To block ATM activation, hNP cells were treated with the ATM inhibitor KU55933 (5 μM, *Sigma‐Aldrich,* St. Louis, MO) for 1 hr before the addition of cisplatin to the culture.

### 4.2 Methods of cell infection with virus shRNA

*Atm* genetic silencing in human NP cells was performed as follows. Human NP cells were seeded on a 6‐well plate at 2 × 10^5^ in 10% F12. The cells were infected at 70% confluency with pLV.ATM or pLV.EGFP with 10% grow medium and polybrene. After an 18‐hr incubation, fresh mediate was added for 4–6 hr, followed by addition of new virus for another 16–24 hr. The cells were cultured for an additional 48–72 hr with 10% growth medium with puromycin (2 ug/mL). The surviving cells were washed with ice‐cold PBS once and then lysed in M‐PER™ Mammalian Protein Extraction Reagent (Thermo Fisher Scientific) with proteinase inhibitors and PhosStop (Roche Diagnostics Corporation; Indianapolis, IN). 20 ug of protein was used on a 4%–2% SDS gel for immunoblotting.

### 4.3 Methods of extract protein from mouse lumber muscle tissue

Mouse lumber muscle tissue was taken from *Ercc1* ^‐/∆^ and Atm^‐/‐^ mice. 20 ug of protein from supernatants was run on 4%–2% SDS gel for immunoblotting.

### 4.4 Mice breeding and isolation of intervertebral discs

Experiments involving mice were approved by the Institutional Animal Care and Use Committee at the University of Pittsburgh (Pittsburgh, PA) and the Scripps Research Institute, Florida. *Ercc1* ^±^ and *Ercc1* ^+/Δ^ mice from C57BL/6J and FVB/n backgrounds, respectively, were crossed to generate *Ercc1^‐/Δ^* F1 hybrid mice to prevent strain‐specific pathology. *Atm^±^* mice were crossed to *Ercc1* ^±^ from C57BL/6J background to generate *Ercc1* ^±^;*Atm^±^*mice, which were then bred with *Ercc1* ^+/Δ^ mice from FVB/n background to generate F1 *Ercc1* ^‐/∆^; *Atm^±^* mice. Breeder mice were backcrossed for 10 generations to achieve genetic homogeneity. Animal protocols used in this study were approved by Scripps Florida Institutional Animal Care and Use Committees.

Nine mice of each of the three strains, WT, *Ercc1* ^‐/∆^, and *Ercc1* ^‐/∆^;*Atm^±^* mice, were sacrificed at 16 weeks of age, and the spines were isolated and dissected under a dissecting microscope. This age was chosen because *Ercc1* ^+/Δ^ mice exhibit pronounced ageing phenotypes by 16 weeks. Entire IVDs were removed *en bloc* from the surrounding vertebral bodies through an incision along the end plates using a surgical number 11 blade. To harvest NP tissue, an axial cut was made on the disc side of the end plate to expose the disc centre, followed by gentle aspiration of the NP tissue, using a sterile P‐10 pipette tip under a dissecting microscope (20–40 × magnification*, Nikon SMZ645; Nikon Instruments Inc.,* Melville, NY).

### 4.5 Cell viability assay

Human nucleus pulposus cells were seeded onto 96‐well plates at 5 × 10^3^ cells per well and cultured for 24 hr. Culture media were then replaced with media containing different cisplatin concentrations (0, 10, 25, 50, 100 µM). Cell viability was assessed using Cell Counting Kit‐8 (CCK‐8) colorimetric assay, which measures the reduction of a tetrazolium salt by dehydrogenases in living cells to a yellow formazan dye product, as per the manufacturer's instructions (*Dojindo, Kumamoto,* Japan). The per cent viability of cells was calculated using the formula: (OD values of treated groups/OD values of untreated control group) × 100%.

### 4.6 1,9‐DMMB colorimetric assay for sulphated glycosaminoglycan

The DMMB colorimetric assay was used to quantify sulphated GAG (Davalos et al., 2013) as a measure of total proteoglycan content. Chondroitin‐6‐sulphate (*Sigma C‐8529,* St. Louis, MO) was used as a standard for GAG. DNA concentration of each sample, measured by the PicoGreen assay (*Molecular Probes,* Grand Island, NY), was used to normalize the GAG values. Average values from 6 reaction samples (2 duplicates × 3 hNP cell cultures derived from 3 patients) were calculated and reported with standard errors. For mice, DMMB assays were performed on NP tissue isolated with the aid of a dissecting microscope (Vo et al., 2010). Average values from 6 reaction samples (6 mice) were calculated and reported with standard errors.

### 4.7 Senescence‐associated β‐galactosidase (SA‐β‐gal) staining for senescent cells

Human nucleus pulposus cells from monolayer cultures were washed in PBS, fixed using 2% formaldehyde and 0.2% glutaraldehyde in phosphate‐buffered saline for 5 min and incubated overnight at 37°C (without CO_2_) with X‐gal‐containing reaction mixture (1 mg/ml X‐gal; 40 mM citric acid/sodium phosphate (pH = 6); 5 mM potassium ferrocyanide; 5 mM potassium ferricyanide; 150 mM NaCl and 2 mM MgCl_2_) as described (Dimri et al., 1995). After overnight incubation, cells were washed with PBS and imaged using a bright field microscope (*Nikon, Eclipse TE2000‐U*).

SA‐β‐gal senescence of *Ercc1^−/−^* MEFs was measured using C_12_FDG staining assay. *Ercc1^−/−^* MEFs were passaged 3 times at 20% O_2_ to induce senescence and then seeded at 2000 cells per well in black wall, clear‐bottom 96‐well plates at least 6 hr prior to treatment. Following the addition of drugs, the MEFs were incubated for 48–72 hr at 20% O_2_. After removing the medium, cells were incubated in 100 nM Bafilomycin A1 in culture medium for 60 min to induce lysosomal alkalinization, followed by incubation with 20 μM fluorogenic substrate C_12_FDG (Setareh Biotech, USA) for 2 hr. Subsequently, cells were washed with PBS and fixed in 2% paraformaldehyde for 15 min and then counterstained with 2 μg/ml Hoechst 33,342 (Thermo Fisher, USA) for 15 min. Finally, cells were imaged with 4–6 fields per well using a high‐content fluorescent image acquisition and analysis platform Cytation 1 (BioTek, VT, USA).

### 4.8 Quantitative reverse transcription–polymerase chain reaction (qRT–PCR)

Total RNA was extracted from cells using TRIzol reagent (Thermo Fisher, USA). cDNA was synthesized using High‐Capacity cDNA Reverse Transcription Kit (Thermo Fisher, USA). Quantitative PCRs were performed with FastStart Universal SYBR Green Master (Rox) from Roche. The experiments were performed according to the manufacturer's instructions. PCR primers used in the study are provided in Table 1.

**Table 1:** Primers used for RT–PCR analysis of gene expression

Gene	Forward (5’—3’)	Reverse (5’—3’)
*GAPDH*	AAGGTCATCCCAGAGCTGAA	CTGCTTCACCACCTTCTTGA
*p16^INK4a^*	CCCAACGCCCCGAACT	GCAGAAGAGCTGCTACGTGAA
*p21 ^Cip1^*	GTCAGGCTGGTCTGCCTCCG	CGGTCCCGTGGACAGTGAGCAG
*IL−1β*	TGGACCTTCCAGGATGAGGACA	GTTCATCTCGGAGCCTGTAGTG
*TNFα*	GGTGCCTATGTCTCAGCCTCTT	GCCATAGAACTGATGAGAGGGAG

### 4.9 Immunoblot analysis

After treatment, cells were washed twice with PBS and lysed on ice for 30 min with whole‐cell extract lysis buffer (*Santa Cruz Biotechnology*, Santa Cruz, CA, USA). 20 µg total protein of each sample was resolved on the 4%–12% gradient SDS‐PAGE and transferred onto PVDF membrane (*Bio‐Rad*, USA) at 350 mA for 2 hr. For ATM detection, 3%–8% gradient SDS‐PAGE was used and PVDF membrane was transferred at 30 V for 16 hr to optimize the transfer of the large‐sized ATM protein (MW ~ 350kD). The membrane‐containing transferred protein was blocked with 5% nonfat milk in Tris‐buffered saline with Tween‐20 (TBST) for 1 hr. The membrane was probed overnight at 4°C with one of the primary antibodies: aggrecan (ab36861*, Abcam)*; p53 (CST 2,524*, Cell Signaling Technology)*; p21*(*sc‐397*, Santa Cruz)*; γH_2_AX *(*05–636*, Millipore)*; ATM *(*ab2618*, Abcam)*; p‐ATM *(*ab81292*, Abcam)*; NF‐κB p65 (sc‐372, *Santa Cruz)*; p‐p65 *(*CST 3,033*, Cell Signaling Technology)*; β‐actin (ab8226, *Abcam*). Quantitation of protein bands was performed by densitometry analysis and local background subtraction using the ChemiDoc^TM^ MP system and its associated Image Lab 5.2.1 Software (*Bio‐Rad*, USA).

### 4.10 Immunofluorescence

Human nucleus pulposus cells were seeded onto glass coverslips, fixed in 4% paraformaldehyde for 15 min and treated with 0.2% Triton X‐100/phosphate‐buffered saline (PBS) for 15 min. Nonspecific binding was blocked by incubation in 5% BSA in PBS. Cells were incubated at 4°C overnight with primary antibody, washed and followed by incubation with the Alexa Fluor 488‐ or Alexa Fluor 647‐conjugated antibody (1:500 dilution, *Invitrogen,* Carlsbad, CA) for 1 hr at 37°C. Finally, cells were mounted using Prolong Gold Antifade reagent with DAPI (*Invitrogen*). Mouse disc tissues were fixed in 10% formalin for 24 hr and then transferred to 30% sucrose in PBS overnight at 4°C. The tissues were frozen in 2‐methylbutane and embedded in optimal cutting temperature at −20°C.

Immunofluorescence was done to detect p21 (sc‐397*, Santa Cruz*) and HMGB1 (ab18256*, Abcam*) proteins on 5‐µm sagittal tissue sections and p16^INK4A^ (ab189032*, Abcam*), and cleaved caspase 3 (Asp175, *Cell Signaling Technology*) to detect senescent and apoptotic cells on hNP cell‐seeded plates. Samples were imaged under a confocal fluorescence microscope (*Nikon Eclipse Ts100; Nikon Instruments Inc.,* Melville, NY). Images used for comparisons of different treatments were acquired using the same instrument settings and exposure times and were processed consistently the same way. The percentages of immunopositive cells for p16^INK4A^ and cleaved caspase 3‐positive hNP cells were calculated.

### 4.11 Measurements of reactive oxygen species (ROS)

The level of cytosolic ROS was quantified with an oxygen radical‐sensitive probe, 2', 7'‐dichlorodihydrofluorescein (H_2_DCFDA). Briefly, hNP cells were treated with 50µM cisplatin for 15 min, washed with PBS and incubated with 10μM H_2_DCFDA (*Invitrogen,* USA) for 30 min in the dark at 37°C. Relative fluorescent intensities were quantified using a flow cytometer using excitation and emission filters of 488 and 530 nm, respectively (*Becton Dickinson,* USA). To measure the level of mitochondria‐specific ROS, 5 μM MitoSOX reagent (*Invitrogen*, USA) was applied to hNP cells grown on the coverslips for 15 min at 37°C protected from light. After repeated washing with warm HBSS, cell nuclei were counterstained by incubation for 10 min with Hoechst 33,342 (1 mg/ml in PBS solution) at room temperature. ROS‐specific fluorescence was assessed using confocal fluorescence microscopy (*Nikon Eclipse Ts100; Nikon Instruments Inc.,* Melville, NY).

### 4.12 Histological Staining

Isolated spines and adjacent vertebral bodies were fixed in 4% paraformaldehyde, decalcified, dehydrated, cleared with dimethylbenzene and embedded in paraffin (*Tissue Tek processor and Leica embedder,* Buffalo Grove, IL). Sections (5 µm) were stained with either haematoxylin and eosin or safranin O (*Fisher Scientific,* Pittsburgh, PA) using standard procedures. Images were acquired under 4X and 20X magnification using a light microscopic system (*Nikon, Eclipse TE2000‐U*).

### 4.13 Statistical analysis

Data were presented as means ± SE (standard error) from 3 to 6 independent experiments. Multiple comparisons of data among the groups were assessed by one‐way ANOVA followed by the least significant difference test (Fisher test). Significance was evaluated by the unpaired Student's *t* test for comparisons between two groups. A *p*‐value < 0.05 was considered statistically significant.

## CONFLICT OF INTEREST

None declared.

## AUTHOR CONTRIBUTIONS

NV, GS, JK, PR and LN formulated the study design. NV, HS, GS, JK, PR and LN obtained funding to support this study. YH, JT, QD, SM, JZ and LZ involved in experimentation and data collection. YH and NV analysed the data. YH, NV, HS, GS, JK, PR and LN interpreted the data and prepared the manuscript. All authors reviewed and approved the manuscript.
